# Chitosan-Based Nanocarriers Co-Delivering Pioglitazone and Curcumin: Biological Activity and Therapeutic Potential in Diabetes

**DOI:** 10.3390/ijms27136002

**Published:** 2026-07-03

**Authors:** Florentina-Geanina Lupascu, Gabriela-Dumitrița Stanciu, Bianca-Ștefania Profire, Roxana-Georgiana Taușer, Dan Lupașcu, Andreea-Teodora Iacob, Ioana-Mirela Vasincu, Maria Apotrosoaei, Alexandru Sava, Bogdan-Ionel Tamba, Lenuța Profire

**Affiliations:** 1 Department of Pharmaceutical and Therapeutical Chemistry, Faculty of Pharmacy, Grigore T. Popa University of Medicine and Pharmacy Iasi, 16 Universitatii Street, 700115 Iasi, Romania; florentina-geanina.lupascu@umfiasi.ro (F.-G.L.); roxana.tauser@umfiasi.ro (R.-G.T.); dan.lupascu@umfiasi.ro (D.L.); andreea.panzariu@umfiasi.ro (A.-T.I.); ioana-mirela.vasincu@umfiasi.ro (I.-M.V.); apotrosoaei.maria@umfiasi.ro (M.A.); lenuta.profire@umfiasi.ro (L.P.); 2 Advanced Center for Research and Development in Experimental Medicine “Prof. Ostin C. Mungiu”, Grigore T. Popa University of Medicine and Pharmacy Iasi, 16 Universitatii Street, 700115 Iasi, Romania; gabriela-dumitrita.stanciu@umfiasi.ro; 3 Department of Internal Medicine, Faculty of Medicine, Grigore T. Popa University of Medicine and Pharmacy Iasi, 16 Universitatii Street, 700115 Iasi, Romania; bianca-stefania.profire@umfiasi.ro; 4 Department of Analytical Chemistry, Faculty of Pharmacy, Grigore T. Popa University of Medicine and Pharmacy Iasi, 16 Universitatii Street, 700115 Iasi, Romania; alexandru.i.sava@umfiasi.ro

**Keywords:** polymer nanoparticle, diabetes mellitus type 2, curcumin, pioglitazone, co-delivery nanocarriers, antioxidant activity, liver toxicity, renal injury, lipidic profile

## Abstract

Diabetes mellitus (DM) is a highly prevalent metabolic disorder and a major public health concern. Pioglitazone, a widely used antidiabetic agent, exhibits limited therapeutic efficiency due to poor water solubility and suboptimal pharmacokinetic properties. Similarly, curcumin (Cur), a natural polyphenol with pleiotropic biological activities, is hindered by low oral bioavailability. In this study, chitosan-based nanocarriers were developed for the delivery of pioglitazone (CS-Pio NPs), curcumin (CS-Cur NPs), and their co-encapsulation (CS-Pio-Cur NPs), aiming to enhance their biological performance and therapeutic efficacy. The co-loaded nanosystem (CS-Pio-Cur NPs) demonstrated significantly enhanced antioxidant activity, as evidenced by DPPH (71.29 ± 0.09%), ABTS (86.08 ± 0.04%), and hydroxyl radical scavenging (87.08 ± 0.06%) assays, along with a strong reducing capacity (IC_50_ = 25.39 ± 0.23 μg/mL). In a diabetic rat model, CS-Pio-Cur NPs significantly reduced blood glucose level and HbA1c (6.60 ± 0.83%), while also improving liver and kidney function parameters and lipid profile. These findings suggest that co-delivery of Pio and Cur via CS-based nanocarriers provides a combined therapeutic effect by simultaneously targeting hyperglycemia, oxidative stress, and associated metabolic dysfunctions. This nanosystem represents a promising approach for improving the management of DM and its complications.

## 1. Introduction

Diabetes mellitus (DM) is a multifactorial metabolic disorder characterized by hyperglycemia arising from defects in insulin secretion, insulin action, or both, and is associated with significant disturbances in carbohydrate, lipid, and protein metabolism [1,2].

DM has been recognized as a major global public health problem by the World Health Organization (WHO) due to its high morbidity, mortality, and associated socioeconomic burden. The incidence of DM has climbed progressively over the last several decades, from around 5% worldwide in 1980 to 10.5% in 2024, according to the International Diabetes Federation. An increase to 12.2% in 2045 is projected, corresponding to an estimated 783 million people affected worldwide [3]. According to the WHO, DM is classified in several categories, such as type 1 diabetes (T1DM), type 2 diabetes (T2DM), gestational diabetes, hybrid forms, and other specific types [4].

T1DM results from autoimmune destruction of pancreatic β-cells leading to insulin deficiency and accounts for 5–10% of diabetes cases, whereas T2DM, representing 90–95% of cases, is primarily characterized by insulin resistance (IR) and progressive β-cell dysfunction [5,6]. The interaction between IR and β-cell dysfunction exacerbates the impairment of glucose homeostasis, resulting in persistent hyperglycemia. In the context of obesity and dyslipidemia, these alterations contribute to the progression and exacerbation of T2DM through interconnected pathophysiological mechanisms. In particular, ectopic lipid accumulation promotes lipotoxicity, oxidative stress, and endoplasmic reticulum stress, which triggers a downstream inflammatory response mediated by sustained cytokine release. Mitochondrial malfunction, characterized by the inadequate oxidation of free fatty acids, concurrently leads to the overproduction of reactive oxygen species (ROS). Together, these processes create a chronic, self-sustaining loop that promotes IR and progressive β-cell depletion [6,7,8].

The development of innovative, more targeted, and selective therapeutic strategies capable of addressing the multifactorial pathophysiology of T2DM remains a major research challenge. These strategies should extend beyond traditional glycemic management and target associated metabolic disorders, including obesity and hyperlipidemia, by modulating critical pathological pathways such as chronic inflammation, oxidative stress, and mitochondrial dysfunction [9]. In this context, the integration of conventional hypoglycemic agents with compounds exhibiting antioxidant and anti-inflammatory properties, alongside their incorporation into advanced nanotechnology-based drug delivery systems, represents a promising approach to enhance therapeutic efficacy and better address the complex nature of T2DM [10].

Polymeric nanoparticles (NPs) have developed as versatile drug delivery systems aiming to improve drug bioavailability, facilitate site-specific targeting, and offer a controlled and sustained release profile, thereby supporting the development of personalized therapeutic strategies [11]. Among these, NPs based on chitosan (CS) have attracted considerable attention due to their biocompatibility, biodegradability, low toxicity properties, and their Generally Recognized As Safe (GRAS) status for selected applications, as well as their extensive use in FDA-regulated pharmaceutical and biomedical fields, including drug delivery and tissue engineering [12]. CS is a naturally derived, cost-effective biopolymer obtained by the deacetylation of chitin, exhibiting excellent mucoadhesive properties. These properties are primarily attributed to electrostatic interactions between its protonated amino groups and the negatively functional groups of mucin that cover the gastrointestinal (GI) epithelium. Consequently, CS-based NPs enhance oral drug delivery by prolonging GI residence time and facilitating both transcellular (via epithelial uptake) and paracellular transport through the reversible opening of tight junctions. These mechanisms collectively contribute to sustained drug release and improved intestinal permeability following oral administration [12,13]. In addition to its function as a biocompatible and mucoadhesive drug delivery carrier, CS exhibits notable antioxidant and anti-inflammatory properties. Its antioxidant potential is mainly attributed to the presence of reactive amino and hydroxyl groups capable of scavenging free radicals and chelating metal ions, thereby reducing oxidative stress. Modulation of the inflammatory response by CS takes place by reducing the regulation of pro-inflammatory mediators and cytokines involved in chronic metabolic disorders. Given that oxidative stress and inflammation are key contributors to diabetes progression and its complications, CS may provide additional therapeutic benefits beyond drug delivery in such pathological conditions [14,15].

Pioglitazone (Pio) is an orally administered thiazolidinedione and a synthetic agonist for peroxisome proliferator-activated receptor gamma (PPARγ), which regulates the transcription of genes associated with glucose and lipid metabolism, thus enhancing insulin sensitivity in adipose tissue, skeletal muscle, and liver. This approach improves peripheral glucose uptake and reduces hepatic gluconeogenesis, contributing to glycemic control in T2DM patients [16]. However, despite its therapeutic advantages, Pio is classified as a Biopharmaceutics Classification System (BCS) class II medication, defined by poor water solubility and high membrane permeability, which limits its oral dissolution rate and, consequently, its oral bioavailability. Moreover, its short half-life (3–6 h), rapid systemic clearance, and variability in GI absorption, especially when food is present, may reduce its pharmacological efficacy and contribute to interindividual variability in treatment response [17,18]. Considering all these challenges associated with Pio, there is an urgent need for the development of improved drug delivery systems designed to enhance solubility, bioavailability, and overall therapeutic performance of Pio, hence increasing its effectiveness in T2DM management.

Curcumin (Cur), a primary curcuminoid obtained from *Curcuma longa*, is a lipophilic polyphenolic compound that has been widely investigated for its broad pharmacological potential [19,20]. Growing evidence highlights its beneficial effects on T2DM, primarily through the modulation of key molecular pathways related to oxidative stress, inflammation, lipid metabolism, and insulin sensitivity [21,22]. Cur specifically exhibits anti-inflammatory effects by blocking pro-inflammatory signaling pathways such as NF-κB and JNK, consequently reducing the production of cytokines including TNF-α, IL-6, and IL-1β, which are associated with the development of IR [21,23]. In addition, Cur has been shown to mitigate oxidative stress by enhancing endogenous antioxidant defenses (e.g., superoxide dismutase, catalase, and glutathione peroxidase) and reducing lipid peroxidation, as indicated by decreased malondialdehyde levels [21,24]. Moreover, Cur enhances lipid homeostasis through the modulation of key metabolic regulators, including AMPK and PPARγ signaling pathways, resulting in decreased triglyceride levels and suppression of hepatic lipogenesis [21,25]. Despite these promising pharmacological properties, the therapeutic use of Cur remains limited due to its poor water solubility, low oral bioavailability, fast metabolism, and chemical instability in physiological conditions. These limitations significantly impair its systemic distribution and therapeutic effectiveness [26,27]. Consequently, considerable research efforts have focused on the development of advanced drug delivery systems, such as polymeric NPs, lipid-based carriers, and inclusion complexes, to enhance their bioavailability and pharmacokinetic profile [28,29].

The aim of this study was to evaluate the biological performance of CS-based NPs, namely CS-Pio NPs, CS-Cur NPs, and the co-loaded CS-Pio-Cur NPs, in comparison with their corresponding free active pharmaceutical ingredients (APIs: Pio, Cur) and their physical mixture (Pio-Cur). Particularly, the antioxidant effects, using in vitro assays, and antidiabetic efficacy in a streptozotocin (STZ)-induced diabetic rat model, were evaluated.

In addition to glycemic control, a panel of complementary biomarkers, including hematological parameters, biochemical indicators of hepatic and renal function, and lipid profile analysis, was evaluated to provide an integrated assessment of metabolic and systemic responses under diabetic conditions. This study builds upon our previous work, which reported the formulation, physicochemical characterization, and in vitro release behavior of these CS-based nanocarrier systems [30].

## 2. Results

### 2.1. Antioxidant Activity

#### 2.1.1. Ferric Reducing Antioxidant Power Assay (FRAP)

**Figure 1:**
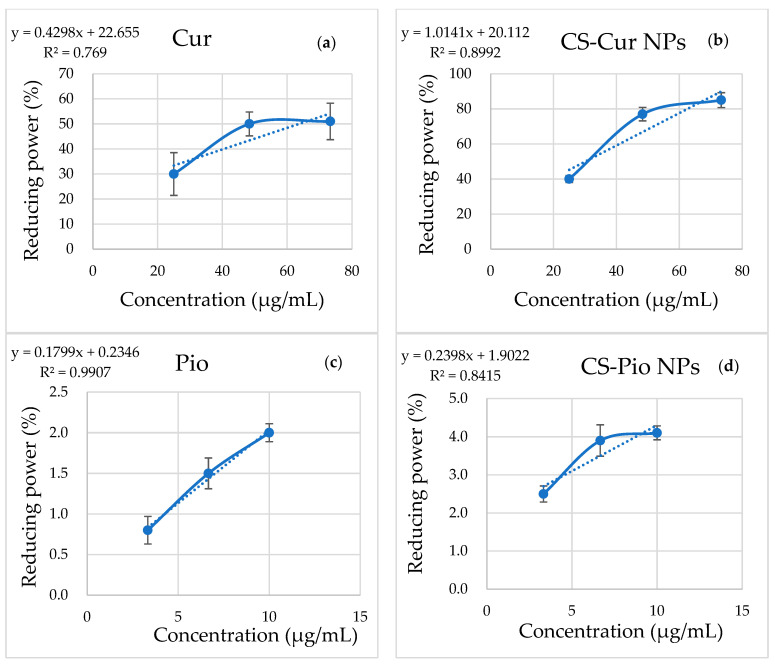

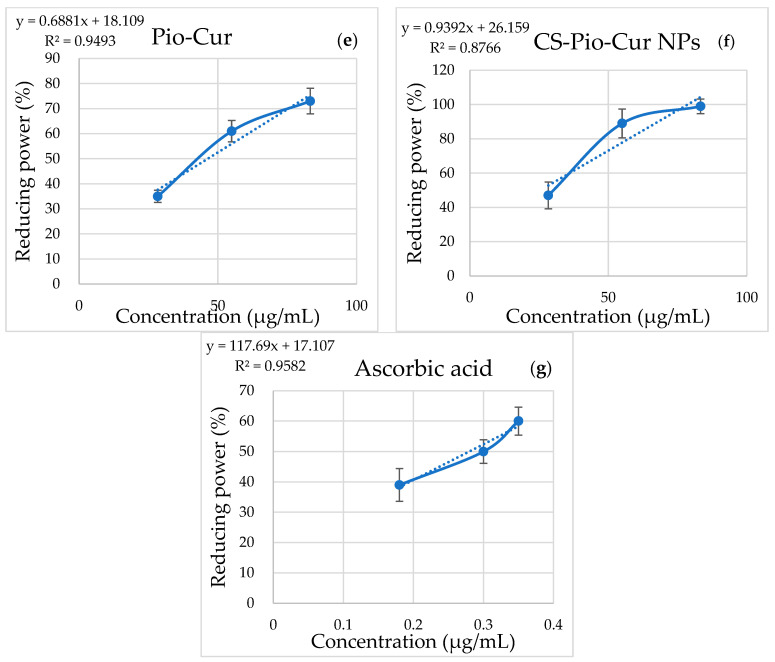
Concentration-response curves of antioxidant capacity determined by the FRAPs of CS-based NPs, APIs (Pio, Cur, Pio-Cur), and ascorbic acid, as a reference standard. APIs: Cur (**a**), Pio (**c**), Pio-Cur (**e**); CS-APIs NPs: CS-Cur NPs (**b**), CS-Pio NPs (**d**), CS-Pio-Cur NPs (**f**); ascorbic acid (**g**). The results are expressed as mean ± SD (*n* = 3).

The ferric reducing antioxidant capacity, expressed as IC_50_ values (µg/mL), was determined from concentration-response curves obtained for CS-based NPs (CS-Pio NPs, CS-Cur NPs, CS-Pio-Cur NPs) and their corresponding APIs (Pio, Cur, Pio-Cur) (Figure 1). The calculated IC_50_ values are summarized in Table 1.

Lower IC_50_ values are indicative of higher antioxidant activity, reflecting an enhanced capacity of the tested compounds to act as reducing agents by converting Fe^3+^ to Fe^2+^. Based on the IC_50_ value, the antioxidant activity of compounds can be classified as follows: very strong (<50 μg/mL), strong (50–100 μg/mL), moderate (101–150 μg/mL), weak (151–200 μg/mL), and inactive (>200 μg/mL) [31].

The analysis of the results demonstrated that encapsulation of APIs within the CS matrix significantly enhanced antioxidant activity, as evidenced by lower IC_50_ values for CS-based NPs formulations (CS-Pio, CS-Cur, CS-Pio-Cur) compared with their corresponding free forms (Pio, Cur, Pio-Cur). The most pronounced improvements were observed for CS-Cur (29.59 ± 0.09 μg/mL) *vs* Cur (59.54 ± 0.20 μg/mL) and for the co-loaded CS-Pio-Cur formulation (25.39 ± 0.23 μg/mL) compared with the physical mixture Pio-Cur (46.34 ± 0.16 μg/mL).

The co-loaded CS–Pio–Cur NPs formulation exhibited the highest antioxidant activity, markedly exceeding that of the single-loaded systems (CS–Cur NPs: 29.59 ± 0.09 μg/mL; CS–Pio NPs: 200.57 ± 0.91 μg/mL). With an IC_50_ value of 25.39 ± 0.23 μg/mL, this formulation can be classified within the “very strong” antioxidant category.

#### 2.1.2. Radical Scavenging Activity

**Figure 2:**
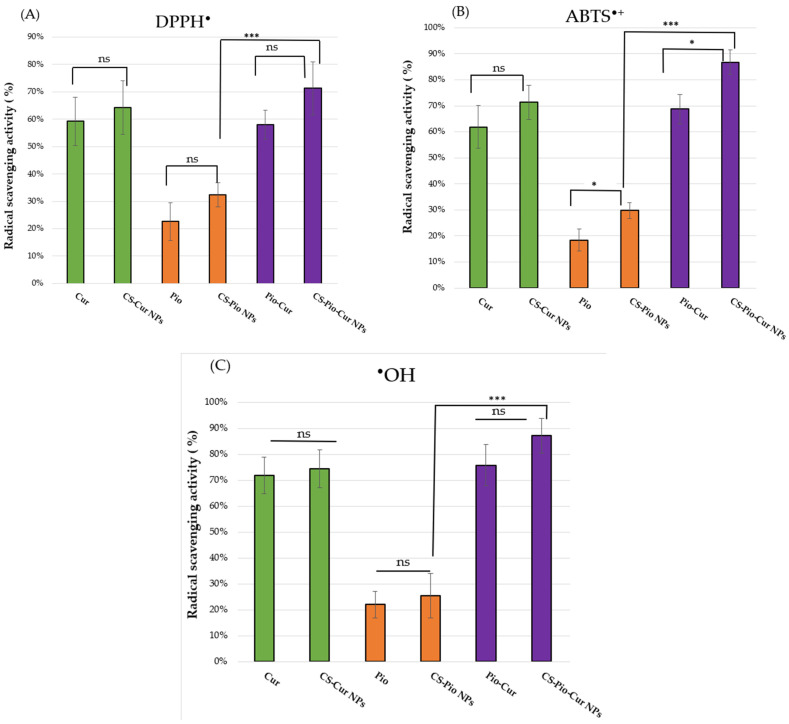
Radical scavenging effects of CS-APIs NPs (CS-Cur NPs, CS-Pio NPs, CS-Pio-Cur NPs) and APIs (Cur, Pio, Pio-Cur) against DPPH^•^ (**A**), ABTS^•+^ (**B**), and •OH (**C**) radicals. The results are expressed as mean ± SD (*n* = 3). Statistical analysis: ns *p* > 0.05, * *p* < 0.05; *** *p* < 0.001.

The results obtained from the three complementary radical scavenging assays (DPPH^•^, ABTS^•+^, and •OH scavenging) (Figure 2) demonstrated a consistent trend, indicating that CS-based NPs (CS-Pio NPs, CS-Cur NPs, and CS-Pio-Cur NPs) exhibited significantly higher radical scavenging activity compared to the corresponding APIs (Pio, Cur, and Pio-Cur). This enhancement can be attributed to the CS matrix, which improves the apparent solubility and dispersion of encapsulated APIs and may also contribute directly to antioxidant activity through its functional groups (hydroxyl and amino groups), capable of interaction with ROS [32].

Among the tested formulations, the co-loaded CS–Pio–Cur NPs exhibited the highest antioxidant activity across all assays. Specifically, their scavenging capacity against DPPH^•^, ABTS^•+^, and •OH radicals was 71.29 ± 0.09%, 86.08 ± 0.04%, 87.08 ± 0.06%, respectively, much higher than the Pio-Cur mixture, for which the values obtained were 57.99 ± 0.05%, 68.75 ± 0.05%, 75.75 ± 0.08%. These findings suggest a combined effect between Pio and Cur when co-encapsulated within the CS-based nanocarrier system.

On the other hand, the co-loaded CS–Pio–Cur NPs exhibited superior scavenging capacity by all three methods compared to both CS-Pio NPs and CS-Cur NPs formulations, the greatest difference being against •OH radicals. Thus, the values obtained were 87.08 ± 0.06% for CS-Pio-Cur NPs, whereas CS-Pio NPs and CS-Cur NPs showed values of 25.41 ± 0.08% and 74.40 ± 0.07%, respectively.

### 2.2. In Vivo Biological Evaluation

#### 2.2.1. Antidiabetic Effects

##### Blood Glucose

T2DM is characterized by chronic hyperglycemia resulting from pancreatic β-cell dysfunction and impaired insulin-mediated glucose uptake in peripheral tissues [33].

**Figure 3:**
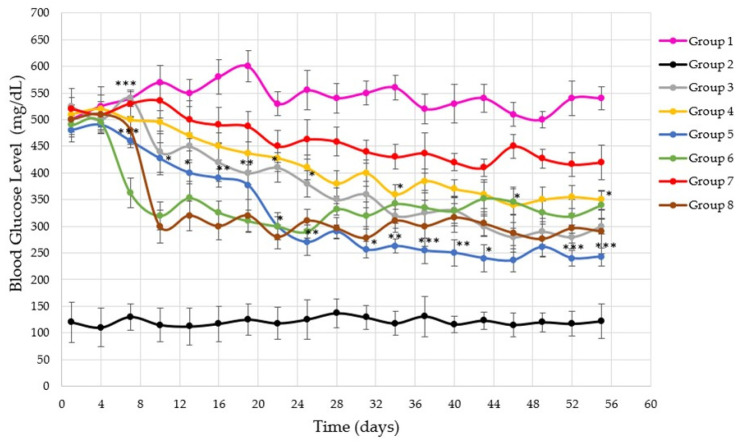
Blood glucose level of the diabetic rats (Group 1), non-diabetic rats (Group 2), diabetic rats treated with CS-Pio NPs (Group 3), CS-Cur NPs (Group 4), CS-Pio-Cur NPs (Group 5), Pio (Group 6), Cur (Group 7), Pio-Cur (Group 8). Data are presented as mean ± SD (*n* = 5). Statistical analysis was performed using two-way repeated measures ANOVA with Geisser–Greenhouse correction, followed by Dunnett’s multiple comparisons test for comparisons versus diabetic control and Šídák’s multiple comparisons test for selected pairwise comparisons between treatment groups. Statistical significance was defined as * *p* < 0.05, ** *p* < 0.01, *** *p* < 0.001.

Diabetic rats exhibited a significant elevation in blood glucose levels compared with non-diabetic rats, confirming the successful induction and maintenance of the diabetic state throughout the experimental period. Chronic administration (55 days) of CS-based nanoformulations (CS–Pio NPs, CS–Cur NPs, and CS–Pio–Cur NPs) and their corresponding free active compounds (Pio, Cur, and Pio–Cur) resulted in a significant reduction in blood glucose levels compared with diabetic rats (Group 1), as demonstrated by the time-dependent changes observed throughout the study period (Figure 3).

Two-way repeated measures ANOVA revealed a significant effect of treatment, time, and treatment × time interaction on blood glucose levels (*p* < 0.0001), indicating that both CS-based nanoformulations and their corresponding free active compounds produced distinct glycemic responses over time. CS-Pio NPs significantly reduced blood glucose levels compared with free Pio, with significant differences observed at days 7, 19, 22, and 25 (*p* = 0.0003, *p* = 0.0075, *p* = 0.0105, and *p* = 0.0306, respectively). Similarly, CS-Cur NPs produced a significantly different glycemic profile compared with free Cur, with differences detected at days 34, 46, and 55 (*p* = 0.0297, *p* = 0.0457, and *p* = 0.0297, respectively). The co-loaded CS-Pio-Cur NPs also showed an improved temporal response compared with free Pio-Cur, with significant differences observed at days 10, 13, and 16 (*p* = 0.0139, *p* = 0.0486, and *p* = 0.0081, respectively).

Furthermore, longitudinal analysis demonstrated that all CS-based nanoformulation treatments produced significant changes in blood glucose levels from baseline to the end of the experiment (day 55), whereas the corresponding free compounds showed weaker or non-significant long-term effects.

Comparative analysis among CS-based nanoformulation treatments revealed differences in their glycemic efficacy. CS-Pio-Cur NPs produced a significantly different glucose-lowering profile compared with CS-Pio NPs, with significant differences observed at days 7, 25, and 31 (*p* = 0.0003, *p* = 0.0262, and *p* = 0.0227, respectively). Moreover, CS-Pio-Cur NPs showed a markedly improved response compared with CS-Cur NPs, with significant differences detected from day 22 and maintained until the end of the experiment (day 55), suggesting a more pronounced and sustained antihyperglycemic effect of the combined nanoformulation.

At the end of the experiment (day 55), one-way ANOVA followed by Tukey’s multiple comparisons test confirmed significant differences among experimental groups.

These results revealed that co-loaded CS–Pio–Cur NPs exerted a markedly enhanced hypoglycemic effect compared to both single-agent therapies and their corresponding free active compound. The co-loaded CS–Pio–Cur NPs demonstrated the most significant decrease in blood glucose levels (49.37 ± 17.34%), exceeding the effects of CS–Pio NPs (42.85 ± 15.21%) and CS–Cur NPs (31.37 ± 18.02%). A similar pattern was observed for the free APIs, where the physical mixture of Pio and Cur produced a pronounced glucose-lowering effect (42 ± 41.21%) compared to Pio alone (30.61 ± 25.70%) and Cur alone (19.23 ± 20.23%), suggesting a favorable interaction between the two agents.

##### Glycated Hemoglobin

HbA1c is a crucial biochemical parameter that reflects long-term glycemic control, indicating the average blood glucose level over approximately 2–3 months. It is often used for monitoring the efficacy of antidiabetic therapy, with values below 7% generally considered indicative of adequate control, according to guidelines from organizations such as the American Diabetes Association [34].

**Figure 4:**
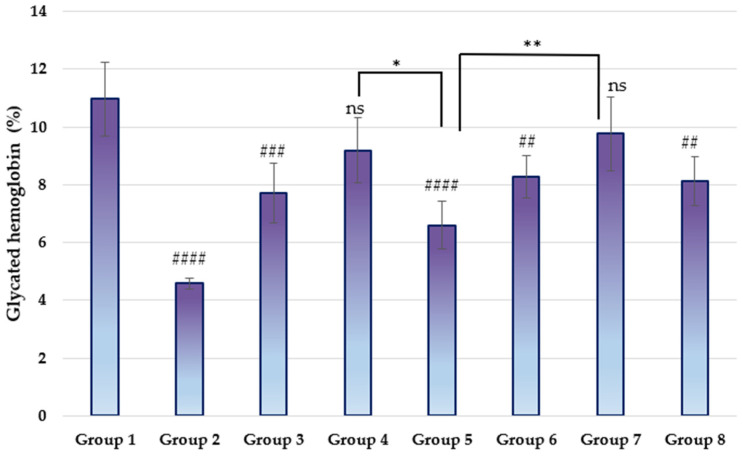
The HbA1c level of the diabetic rats (Group 1), non-diabetic rats (Group 2), diabetic rats treated with CS-Pio NPs (Group 3), CS-Cur NPs (Group 4), CS-Pio-Cur NPs (Group 5), Pio (Group 6), Cur (Group 7), and Pio-Cur (Group 8). The results are expressed as mean ± SD (*n* = 5). Statistical analysis was performed using one-way ANOVA followed by Tukey’s multiple comparisons test. Statistical significance was considered at *p* < 0.05. Comparisons versus diabetic control group (Group 1): ## *p* < 0.01, ### *p* < 0.001, #### *p* < 0.0001. Comparisons between treated groups: * *p* < 0.05 and ** *p* < 0.01. ns indicates no statistically significant difference (*p* > 0.05).

In the present study, one-way ANOVA analysis revealed a significant effect of CS-based nanoformulations (CS–Pio NPs, CS–Cur NPs, and CS–Pio–Cur NPs) and their corresponding free active compounds (Pio, Cur, and Pio–Cur) on HbA1c levels (F(7,32) = 16.42, *p* < 0.0001), confirming significant differences among the experimental groups. Compared with the diabetic rats (Group 1, HbA1c = 10.96 ± 1.26%), all treatment groups (Group 3–8) exhibited a reduction in HbA1c levels (Figure 4).

Among the groups treated with free APIs, the Pio–Cur combination (Group 8) showed a slightly greater reduction in HbA1c compared with Pio (Group 6) and Cur (Group 7), with mean values of 8.13 ± 0.84%, 8.27 ± 0.72%, and 9.76 ± 1.27%, respectively. These findings suggest a trend toward an improved glycemic response following the combined administration of Pio and Cur compared with the individual treatments; however, the observed differences did not reach statistical significance.

Notably, CS-based nanoformulations showed different degrees of improvement in long-term glycemic control. Treatment with CS–Pio NPs (Group 3) and CS–Cur NPs (Group 4) resulted in HbA1c values of 7.71 ± 1.02% and 9.19 ± 1.14%, respectively, while the co-loaded CS–Pio–Cur NPs formulation (Group 5) produced the lowest HbA1c value among all treated groups (6.60 ± 0.83%), approaching the level observed in non-diabetic rats (Group 2, HbA1c = 4.58 ± 0.19%).

Importantly, the CS–Pio–Cur NPs group demonstrated the most favorable glycemic profile among all CS-based NPs and free counterparts, with a significant difference compared with Group 4 and Group 7 (*p* = 0.0131 and *p* = 0.0014, respectively). Although direct comparisons between each CS-based nanoformulation and its corresponding free active compounds did not reach statistical significance for HbA1c, the trend towards lower HbA1c values supports the potential benefit of nanoencapsulation in improving sustained glycemic control. On the other hand, given that HbA1c reflects cumulative glycemic exposure rather than short-term glucose fluctuations, the observed differences suggest that CS-based nanoformulations, especially co-loaded CS-Pio-Cur NPs, may provide improved metabolic control during chronic treatment.

#### 2.2.2. Hematological and Biochemical Measurements

##### Hematological Profile

The hematological profiles of diabetic rats treated with CS-based NPs (CS–Pio NPs, CS–Cur NPs, and CS–Pio–Cur NPs), their corresponding free APIs (Pio, Cur, and Pio-Cur), and alongside diabetic and non-diabetic controls, are summarized in Table 2.

Mean corpuscular volume (MCV), red blood cell count (RBC), hematocrit (HCT), red cell distribution width (RDW-SD and RDW-CV) showed a remarkable alteration in STZ-induced diabetic rats (Group 1), while the administration of CS-based NPs and free APIs restored the changes in all these parameters, with values close to those recorded in the non-diabetic Group (Group 2).

Diabetic rats showed a significant increase in inflammatory and immune system markers, including white blood cell count (WBC), lymphocyte percentage (LYM%), and monocyte count (MONO), together with a decreased absolute lymphocyte count (LYM#), reflecting lymphopenia induced by hyperglycemia and stress-related cortisol elevation. This leads to lymphocyte apoptosis and redistribution from peripheral blood, while relative changes in other leukocyte populations contribute to the apparent increase in LYM%. Chronic administration of the CS-based NPs and free APIs resulted in a slight restoration of these inflammatory parameters, particularly in rats treated with CS-Pio-Cur NPs, with values approaching those observed in the non-diabetic Group (Group 2).

Platelet count (PLT) and proportion of large and metabolically active platelets (P-LCR) showed increased values in the diabetic Group compared to the non-diabetic Group, suggesting a platelet activation associated with hyperglycemic status. After administration of the investigated compounds, PLT and P-LCR values showed a tendency to normalize, which is more pronounced in the Group treated with CS-Pio-Cur NPs (Group 5), with the differences being statistically significant (*p* < 0.05).

##### Lipid Profile

Analysis of lipid profile parameters was performed to assess the potential modulatory effects of the tested formulations on diabetes-associated dyslipidemia in rats. The parameters analyzed included total cholesterol (TC), triglycerides (TG), HDL-cholesterol, LDL-cholesterol, and VLDL-cholesterol.

Diabetic rats (Group 1) exhibited significant dyslipidemia (*p* < 0.05), characterized by increased levels of TG, LDL, TC, and VLDL, along with a marked decrease in HDL, compared with non-diabetic rats (Group 2) (Table 3).

Treatment of diabetic rats with CS-based nanoformulations (CS-Pio NPs, CS-Cur-NPs, CS-Pio-Cur NPs) and free APIs (Pio, Cur, Pio-Cur) significantly attenuated these alterations in lipid profile parameters compared with diabetic rats (Group 1). Notably, the most pronounced effect was observed in the Group treated with CS–Pio–Cur NPs (Group 5), which demonstrated substantial reductions in TG, TC, VLDL, and LDL levels compared to diabetic rats (Group 1). Moreover, HDL levels in this Group (37 ± 3.41 mg/dL) were restored to values comparable to those of non-diabetic rats (38 ± 2.11 mg/dL), indicating a near-complete normalization of lipid homeostasis.

##### Liver Function

Liver enzyme (aspartate aminotransferase—AST, alanine aminotransferase—ALT, lactate dehydrogenase—LDH) and liver function biomarker (total bilirubin—TB, direct bilirubin—DB) evaluation was performed to determine the modulatory effects of the tested formulations on diabetes-associated liver failure in rats.

Treatment of diabetic rats with CS-based nanoformulations (CS-Pio NPs, CS-Cur NPs, and CS-Pio-Cur NPs) and free APIs (Pio, Cur, and Pio-Cur) significantly reduced the levels of the investigated biochemical parameters, suggesting a protective effect against STZ- and diabetes induced liver dysfunction compared with diabetic rats (Group 1). These findings suggest a hepatoprotective effect of Cur, likely mediated by its antioxidant and anti-inflammatory properties. It was noted that the co-loaded CS-Pio-Cur NPs showed the most pronounced protective effect, significantly (*p* < 0.05) reducing ALT, AST, LDH, and bilirubin levels, with values approaching those of the non-diabetic Group (Table 4).

##### Renal Function

Analysis of renal function biomarkers (creatinine, uric acid, blood urea nitrogen—BUN) was conducted to assess the modulatory effects of the tested formulations on STZ-induced renal dysfunction in diabetic rats.

The findings (Table 5) indicated that diabetic rats (Group 1) exhibited significant renal impairment, as evidenced by elevated serum levels of BUN, creatinine, and uric acid, in comparison to non-diabetic rats (Group 2).

Treatment with CS-based nanoformulations, particularly the co-loaded CS–Pio–Cur NPs (Group 5), resulted in a marked reduction in renal function biomarkers, exhibiting values approximately twofold lower than those observed in the diabetic Group (Group 1) and approaching the levels observed in the non-diabetic Group (Group 2).

## 3. Discussion

DM remains a major global health challenge worldwide due to its increasing prevalence and significant clinical and socioeconomic implications [35]. Although multiple pharmacological approaches are currently available, their use has been limited by adverse reactions, reduced bioavailability, and suboptimal therapeutic responses. To overcome these limitations, nanotechnology-based controlled drug delivery systems have emerged as an attractive alternative for improving therapeutic outcomes.

The present study aimed to evaluate CS-based nanoformulations (CS-Pio NPs, CS-Cur NPs, and CS-Pio-Cur NPs) as potential therapeutic systems for improving glycemic control and attenuating oxidative stress, inflammation, and diabetes-related metabolic disturbances in STZ-induced diabetic rats. To comprehensively investigate their therapeutic efficacy, both in vitro antioxidant activity and in vivo antidiabetic effects were assessed by analyzing glycemic parameters, hematological profiles, biochemical markers of hepatic and renal function, and lipid profile parameters.

The study of antioxidant effects (DPPH^•^, ABTS^•+^, and •OH radical scavenging and FRAPs) revealed that CS-API formulations had superior antioxidant activity compared to their free counterparts. The observed improvement can be attributed to the combined effects of the intrinsic antioxidant capacity of Cur, the contribution of functional groups in the CS matrix (hydroxyl and amino groups) [32], and the potential combined interactions between the co-encapsulated agents (Cur and Pio). Among all CS-API formulations, the co-delivery system showed the highest capacity to interact with ROS; the highest scavenging activity was against •OH, followed by ABTS^•+^ and DPPH^•^. It is known that among ROS, •OH radicals are the most reactive species and are capable of inducing extensive cellular damage through interactions with lipids, proteins, and nucleic acids, ultimately contributing to oxidative stress and apoptosis [36]. Therefore, efficient elimination of these species is essential for cellular protection.

In the in vivo STZ-induced diabetic model, CS-API formulations demonstrated a superior decrease in blood glucose levels compared to the corresponding physical mixture, highlighting the role of the CS-based nanocarrier system in enhancing therapeutic performance. Among all formulations, CS–Pio–Cur NPs exerted the most pronounced hypoglycemic effect, significantly reducing blood glucose and HbA1c levels to values approaching those of the non-diabetic rat Group. The higher effectiveness of the CS–Pio–Cur formulation likely arises from the combined effects of the two compounds, combined with improved bioavailability and sustained drug release, supporting its potential as an advanced therapeutic strategy for T2DM management.

Given that chronic hyperglycemia is associated with oxidative stress and systemic inflammation leading to the onset of diabetes complications, including hematological alterations, the impact of the tested formulation on the hematological profile of diabetic rats was also evaluated in this study [37].

The hematological profile parameters evaluated were red blood cell count (RBC), hematocrit (HCT), hemoglobin (HGB), mean corpuscular volume (MCV), mean corpuscular hemoglobin concentration (MCHC), and red cell distribution width (RDW-CV and RDW-SD), as well as leukocyte indices (white blood cell count, WBC; lymphocyte percentage LYM%; absolute lymphocyte count LYM#; and monocyte count, MONO) and platelet parameters (platelet count, PLT; platelet large cell ratio P-LCR) [37].

In diabetic conditions, reduced HGB levels are commonly reported and may result from oxidative damage to erythrocytes and impaired erythropoiesis, often exacerbated by renal dysfunction [37]. In our study, HGB and MCHC values remained relatively unchanged in all treated Groups compared to diabetic rats (Group 1), whereas significant alterations (*p* < 0.05) were observed in MCV, RBC, HCT, RDW-SD, and RDW-CV. These findings indicate erythrocyte structural diversity instead of a decrease in hemoglobin content per cell, suggesting early-stage hematological alterations associated with diabetic status.

Leukocyte-related parameters, such as WBC, LYM, LYM#, and MONO, are well-established indicators of inflammation and immune activation. Consistent with other reports, T2DM is associated with a persistent low-grade inflammatory state characterized by increased LYM and modified lymphocyte profiles [38,39,40]. Our study demonstrated that treatment with CS-based NPs and free APIs resulted in a significant reduction (*p* < 0.05) in inflammatory markers, with the most notable normalization observed in the CS-Pio-Cur NPs. LYM# showed a rising trend toward values similar to those in the non-diabetic Group, suggesting a possible return to a balanced immune system. These findings suggest that co-delivery of Pio and Cur, particularly within a CS nanocarrier system, may effectively attenuate diabetes-associated inflammation, possibly through modulation of oxidative stress and cytokine-mediated pathways.

Platelet indices further supported the presence of a pro-inflammatory and pro-thrombotic state in diabetic rats. Increased PLT and P-LCR observed in diabetic rats are consistent with increased platelet activation and turnover, which are associated with an elevated risk of thrombotic complications, including diabetic retinopathy [41,42]. Treatment with CS-Pio-Cur NPs led to a significant decrease in PLT (*p* < 0.05), approaching the levels observed in non-diabetic controls. This effect may be attributed, at least in part, to the anti-inflammatory and anti-platelet aggregation properties of Cur, suggesting a protective role of the co-loaded system against diabetes-associated hematological and vascular complications.

For a comprehensive investigation of the therapeutic potential of the tested formulations, the lipid profile and parameters of liver and kidney function in diabetic rats were evaluated.

Dyslipidemia is a common metabolic disturbance in patients with T2DM, with a reported prevalence ranging from 64% to 87.5%, and is closely associated with inadequate glycemic control, insulin resistance, obesity, and chronic low-grade inflammation [43,44].

The pathophysiology of diabetic dyslipidemia is primarily driven by insulin resistance, which promotes the activation of hormone-sensitive lipase in adipose tissue, leading to increased release of free fatty acids (FFAs) into circulation. The increased influx of FFAs to the liver promotes TG synthesis and VLDL production. At the same time, insulin resistance impairs the activity of lipoprotein lipase, leading to decreased clearance of TG-rich lipoproteins, which therefore results in increased circulating levels of TG and VLD [45]. These pathways lead to the characteristic lipid abnormalities observed in T2DM. Moreover, increased levels of FFAs contribute to enhanced production of ROS, promoting lipid peroxidation, nucleic acid impairment, and cellular dysfunction, which exacerbate endothelial dysfunction and further aggravate insulin resistance [45].

On the other hand, the pathophysiology of T2DM involves several interrelated mechanisms, including hepatic dysfunction characterized by lipid accumulation (hepatic steatosis), oxidative stress, and the development of hepatic IR, leading to increased hepatic glucose production [46]. In addition to diabetes-associated metabolic dysfunction, liver injury, occurring in the STZ-experimental model, induces hepatocellular damage and necrosis. This process is reflected by elevated levels of liver enzymes, including ALT, AST, and LDH, resulting from compromised cell membrane integrity and subsequent enzyme leakage into the circulation. Moreover, compromised hepatic function affects bilirubin metabolism, leading to increased TB levels, often with a predominance of the direct (conjugated) fraction [47].

Diabetic nephropathy is one of the most common metabolic disorders associated with T2DM, characterized by glomerular and tubular dysfunction, ultimately resulting in a reduction in glomerular filtration rate. These alterations are reflected by increased serum levels of creatinine, blood urea nitrogen (BUN), and uric acid, which are essential indicators of compromised renal excretory function [48,49]. These alterations are consistent with the effects of chronic hyperglycemia, which induces oxidative stress, inflammation, and structural damage in renal tissue, leading to compromised renal function.

Diabetic control rats showed pronounced dyslipidemia together with impaired hepatic and renal function, presumably due to STZ-induced toxicity and the metabolic disturbances associated with diabetic pathophysiology. This study demonstrated that CS-based NPs formulations, particularly the co-loaded nanoformulation (CS–Pio–Cur NPs), exhibit an enhanced protective effect on metabolic disturbances induced by diabetes compared to both free APIs (Pio, Cur) and their physical mixture (Pio-Cur). This can be attributed to the increased solubility and bioavailability of APIs dispersed in the CS-based nanocarrier, which potentiates their hypoglycemic, antioxidant, and anti-inflammatory effects, in addition to a potentially combined effect between Pio and Cur. Overall, CS–Pio–Cur NPs represent a promising dual-delivery system with potential for the integrated management of T2DM and its associated complications. Future studies are necessary to clarify the molecular pathways responsible for these effects and to further validate the translational potential of this nanoformulation.

## 4. Materials and Methods

### 4.1. Materials

Chemicals. Curcumin (Cur, ≥94% curcuminoid content; ≥80% curcumin), pioglitazone hydrochloride (Pio, MW = 392.90 g/mol), 2,2-diphenyl-1-picrylhydrazyl (DPPH), 2,2′-Azino-bis (3-ethylbenzothiazoline-6-sulfonic acid) (ABTS), L-ascorbic acid 99%, potassium ferricyanide 99%, trichloroacetic acid ≥99%, ferric chloride hexahydrate 97%, iron(II) sulfate heptahydrate ≥99%, hydrogen peroxide solution (H_2_O_2_, ≥30%), ammonium persulfate ≥98%, streptozocin (≥75% α-anomer basis, ≥98% HPLC) were purchased from Sigma-Aldrich, St. Louis, MO, USA.

*CS-based NPs (CS-Pio NPs, CS-Cur NPs, CS-Pio-Cur NPs).* The physicochemical properties of the CS-API NPs were previously reported [30]. Briefly, the formulations showed suitable particle size (211.6–337.4 nm), low polydispersity (0.104–0.289), positive zeta potential (21.83–32.64 mV), high encapsulation efficiency and loading capacity, and a controlled, prolonged drug-release profile.

### 4.2. In Vitro Antioxidant Assays

The antioxidant efficacy of the developed CS-based NPs (CS-Pio NPs, CS-Cur NPs, CS-Pio-Cur NPs) was assessed through various complementary assays, including the FRAPs and radical scavenging assays against DPPH^•^, ABTS^•+^, and •OH, to provide a comprehensive evaluation of their redox-modulating potential.

#### 4.2.1. Ferric Reducing Antioxidant Power Assay (FRAP)

The FRAP is based on the capacity of antioxidants to function as electron donors, reducing ferric ions (Fe^3+^) to their ferrous form (Fe^2+^). In the presence of reducing agents, the ferric/ferricyanide complex is converted to its ferrous counterpart, which subsequently reacts with ferric chloride to form the Prussian blue (Fe_4_[Fe(CN)_6_]_3_) complex. The intensity of this complex can be quantified spectrophotometrically, at approximately 700 nm, and is directly proportional to the reducing power and antioxidant capacity of the sample [50].

4FeCl_3_ + 3K_4_[Fe(CN)_6_] → Fe_4_[Fe(CN)_6_]_3_ + 12KCl

A quantity of CS-APIs NPs: 4.32 mg of CS-Pio-Cur NPs (containing 0.88 mg Cur and 0.12 mg Pio), 3.21 mg of CS-Cur NPs (containing 0.88 mg Cur), 2.66 mg of CS-Pio NPs (containing 0.12 mg Pio), and APIs (Cur, Pio, Cur-Pio) in the same concentrations were dissolved in 1.5 mL ethanol. Aliquots (0.25–0.75 mL) from each sample were further diluted to a final volume of 1.5 mL with ethanol, and then 1.5 mL of phosphate buffer (0.2 M, pH 6.6) was added. The reaction was initiated by adding 1.5 mL of potassium ferricyanide solution (1% *w*/*v*), followed by incubation at 50 °C for 20 min. After incubation, the reaction was stopped by cooling the sample and adding 1.5 mL of trichloroacetic acid (10% *w*/*v*). The mixtures were then centrifuged at 8.000 rpm for 10 min. After that, an aliquot of 2.5 mL of the supernatant was mixed with 0.5 mL of ultrapure water and 0.5 mL ferric chloride solution (0.1% *w*/*v*). The absorbance of the resulting Prussian blue complex was measured at 700 nm against a blank prepared according to the same protocol but without sample, using a UV-VIS spectrophotometer (UV-2600, Shimadzu Instruments Co., Kyoto, Japan). An increase in absorbance was considered indicative of higher reducing power and, consequently, greater antioxidant capacity. Ascorbic acid (0.18–0.35 µg/mL) was used as a reference standard and subjected to the same protocol. The reducing power was calculated using the following formula [51]:

% Reducing Power = (A_sample_ − A_blank_)/A_sample_ × 100,

where A_sample_ = the absorbance of the sample; A_blank_ = the absorbance of the blank.

The obtained values were used to construct calibration curves by plotting reducing power (%) versus sample concentration (µg/mL), followed by linear regression analysis. The half-maximal inhibitory concentration (IC_50_), defined as the concentration required to achieve 50% reduced activity, was subsequently determined [51]:

IC_50_ = (50 − a)/b

where a = slope; b = intercept.

#### 4.2.2. DPPH Radical Scavenging Assay (DPPH^•^)

The DPPH radical scavenging assay is based on the reduction in the stable free radical 2,2-diphenyl-1-picryl hydrazyl (DPPH^•^) to its non-radical form (DPPH-H), in the presence of antioxidant compounds acting as hydrogen or electron donors. This reaction results in a color change from deep purple to pale yellow, which can be quantitatively monitored spectrophotometrically at 517 nm. A decrease in absorbance is directly proportional to the radical scavenging activity of the tested sample [50,52]. An ethanolic DPPH solution (0.1 mM) was prepared and diluted with ethanol to obtain an initial absorbance of 0.95 ± 1.05, representing the blank control (A_DPPH_).

A quantity of CS-APIs NPs: 4.32 mg of CS-Pio-Cur NPs (containing 0.88 mg Cur and 0.12 mg Pio), 3.21 mg of CS-Cur NPs (containing 0.88 mg Cur), 2.66 mg of CS-Pio NPs (containing 0.12 mg Pio), and APIs (Pio, Cur, Pio-Cur) in the same concentrations were dissolved in 10 mL of ethanol. Subsequently, 10 mL of the DPPH ethanolic solution was added, and the mixtures were incubated for 30 min at room temperature in the dark. After incubation, the absorbance of each sample was measured at 517 nm using a UV-VIS spectrophotometer (UV-2600, Shimadzu Instruments Co., Japan). The DPPH radical scavenging rate (%) was calculated using the following formula [53]:

DPPH (%) = [(A_DPPH_ − A_s_)/A_DPPH_] × 100,

where A_DPPH_ = the absorbance of the blank control; A_s_ = the absorbance of the samples.

#### 4.2.3. ABTS Radical Scavenging Activity Assay (ABTS^•+^)

The ABTS radical scavenging assay is based on the generation of the stable radical cation ABTS^•+^ by reaction of ABTS aqueous solution (7 mM) with ammonium persulfate (2.45 mM) in a 1:1 (*v*/*v*) ratio. The mixture was incubated in the dark for 16 h at room temperature to allow complete formation of the ABTS^•+^ radical. Prior to use, the resulting solution was diluted with ethanol to obtain an absorbance of 0.7 ± 0.05 at 734 nm.

A quantity of CS-APIs NPs: 4.32 mg of CS-Pio-Cur NPs (containing 0.88 mg Cur and 0.12 mg Pio), 3.21 mg of CS-Cur NPs (containing 0.88 mg Cur), and 2.66 mg of CS-Pio NPs (containing 0.12 mg Pio) and APIs (Pio, Cur, Pio-Cur) in the same concentrations were dissolved in 1 mL of ethanol. Subsequently, 4 mL of the ABTS^•+^ solution was added, and the mixtures were incubated for 30 min at room temperature. The absorbance was measured at 734 nm using a UV–Vis spectrophotometer (UV-2600, Shimadzu Instruments Co., Japan). The ABTS radical scavenging rate (%) was calculated according to the following formula [53]:

ABTS (%) = [(A_ABTS_ − A_s_)/A_ABTS_] × 100

where A_ABTS_ = the absorbance of the blank control; As = the absorbance of the samples.

#### 4.2.4. Hydroxyl Radical Scavenging Activity Assay (•OH)

The hydroxyl radical (•OH) scavenging assay is based on the generation of •OH radicals via the Fenton reaction [34,52]. In this reaction, ferrous ions (Fe^2+^) catalyze the decomposition of hydrogen peroxide (H_2_O_2_), producing highly reactive •OH radicals according to the following reaction [36]:

Fe^+2^ + H_2_O_2_ = Fe^+3^ + H_2_O + •OH

The generated •OH radicals react with salicylic acid to form hydroxylated derivatives, primarily 2,3-dihydroxybenzoic acid, which can be quantified spectrophotometrically at 510 nm.

•OH + salycilic acid → OH-salycilic acid adduct^•^

Fe^3+^ + OH-salycilic acid adduct^•^ → Fe^2+^ + salycilic acid^•^ oxidation products

In the presence of antioxidant compounds, the formation of these oxidation products is inhibited, resulting in a decrease in absorbance proportional to the radical scavenging activity [54].

A quantity of CS-APIs NPs: 4.32 mg of CS-Pio-Cur NPs (containing 0.88 mg Cur and 0.12 mg Pio), 3.21 mg of CS-Cur NPs (containing 0.88 mg Cur), 2.66 mg of CS-Pio NPs (containing 0.12 mg Pio), and APIs (Pio, Cur, Pio-Cur) in the same concentrations were dissolved in 2 mL ethanol and kept in the dark for 10 min. Subsequently, 2 mL of FeSO_4_ aqueous solution (4 mM), 2 mL of salicylic acid solution (3 mM, in ethanol), and 2 mL of H_2_O_2_ solution (0.3%, *v*/*v*) were added sequentially. The reaction mixtures were incubated at 37 °C for 15 min. After incubation, the absorbance was measured at 510 nm using a UV-Vis spectrophotometer (UV-2600, Shimadzu Instruments Co., Japan). A blank control (without sample) and a sample control (without FeSO_4_) were prepared under identical conditions.

The hydroxyl radical scavenging rate (%) was calculated according to the following formula [55]:

•OH scavenging activity (%) = (1 − (A_s_ − A_c_)/A_0_) × 100,

where A_s_ = the absorbance of the samples, A_c_ is the absorbance of the sample control, and A_o_ = the absorbance of the blank control.

### 4.3. In Vivo Antidiabetic Effects

#### 4.3.1. Streptozotocin-Induced Diabetes Model

##### Animals

Adult male Wistar rats (200–250 g) were used in this study. The animals were housed in standard laboratory conditions in polypropylene cages, maintained at a controlled temperature (24 ± 2 °C), with a 12 h light/dark cycle, and had free access to standard laboratory chow and water ad libitum. The animals were acclimatized for two weeks prior to the experimental procedure to ensure psychological and behavioral stabilization. All experimental protocols were conducted in accordance with internationally accepted guidelines for the veterinary care of laboratory animals and received approval from the Ethics Committee of the Veterinary Sanitary and Food Safety of Iasi (No. 26/23 October 2020).

##### Experimental Design

T2DM was induced by a single intraperitoneal injection of streptozotocin (STZ, 50 mg/kg), freshly dissolved in citrate buffer (10 mM, pH 4.5). The development of diabetes was confirmed one week post-injection by persistent hyperglycemia (fasting blood glucose > 250 mg/dL), accompanied by classic symptoms such as polyuria and polydipsia. Following confirmation, animals were randomly assigned to experimental groups. The group size was 5 rats (*n* = 5) established based on previous studies using STZ-induced diabetic rat models and in accordance with the reduction principle of animal research, balancing statistical reliability with the ethical requirement to minimize animal use. A group of non-diabetic rats was also formed. No exclusions or mortality were recorded during the 55-day experimental protocol. Therefore, attrition was zero, and all animals were included in the final analysis, being distributed in 8 experimental groups.

**Group 1:** diabetic rats treated with vehicle (Tween 80, 2 mL/kg, daily) for 55 days;

**Group 2:** non-diabetic control rats treated with vehicle (Tween 80, 2 mL/kg daily) for 55 days;

**Group 3**: diabetic rats treated with CS-Pio NPs (equivalent to 5 mg/kg Pio);

**Group 4:** diabetic rats treated with CS-Cur NPs (equivalent to 30 mg/kg Cur);

**Group 5:** diabetic rats treated with CS-Pio-Cur NPs (equivalent to 5/30 mg/kg Pio/Cur);

**Group 6:** diabetic rats treated with Pio (5 mg/kg);

**Group 7:** diabetic rats treated with Cur (30 mg/kg);

**Group 8:** diabetic rats treated with Pio-Cur (5/30/mg/kg).

The CS-based NPs (CS-Pio NPs, CS-Cur NPs, CS-Pio-Cur NPs) and APIs (Pio, Cur, Pio-Cur) were administered once daily by oral gavage, suspended in Tween 80, for a duration of 55 days. Blood glucose levels were monitored every 3 days using a glucometer under fasting conditions.

##### Blood Sample Collection and Biochemical Analysis

At the end of the experiment, blood samples were collected via cardiac puncture under appropriate conditions. Samples were divided into tubes containing anticoagulant and tubes without anticoagulant for serum separation.

Whole blood samples were used for the assessment of hematological parameters and glycated hemoglobin (HbA1c). Serum samples were obtained by centrifugation at 3500 rpm for 15 min and were later used for biochemical analysis, including lipid profile assessment and evaluation of hepatic and renal function markers. All hematological and biochemical parameters were analyzed using an automated biochemistry analyzer (RX Imola, Randox Laboratories, London, UK).

##### Statistical Analysis

Statistical analyses were performed using GraphPad Prism version 11.0 (GraphPad Software, San Diego, CA, USA). Data are expressed as mean ± standard deviation (SD). Comparisons between two experimental groups were performed using Student’s *t*-test. Differences among multiple groups were assessed using one-way analysis of variance (one-way ANOVA) followed by Tukey’s multiple comparisons test. For the analysis of blood glucose levels measured repeatedly during the 55-day experimental period, a two-way repeated measures ANOVA with Geisser–Greenhouse correction was performed, followed by Dunnett’s multiple comparisons test for comparisons against diabetic control and Šídák’s multiple comparisons test for selected pairwise comparisons between treatment groups. A *p*-value < 0.05 was considered statistically significant.

## 5. Conclusions

The present study highlights the potential of CS-based nanoformulations as efficient carriers for the combined delivery of Pio and Cur. Among the investigated formulations, the co-loaded CS–Pio–Cur NPs demonstrated the most favorable overall biological response, supporting the advantages of integrating both active compounds within a single nanocarrier platform.

In the STZ-induced diabetic rat model, administration of the co-loaded nanoformulation was associated with improvements in glycemic control, as well as in several diabetes-related biochemical and hematological parameters. In addition, the formulation exhibited enhanced antioxidant activity, suggesting a potential role in mitigating oxidative stress, a key contributor to the progression of diabetic complications.

Importantly, while the co-loaded CS–Pio–Cur NPs showed more pronounced effects compared to single-agent therapies and free active compounds, these findings should be interpreted with caution, as they are limited to an experimental animal model. Therefore, the observed effects should be considered as preliminary evidence of therapeutic potential rather than definitive proof of clinical efficacy.

Overall, the results indicate that CS–Pio–Cur NPs represent a promising strategy for the co-delivery of antidiabetic and antioxidant agents. However, further studies are required to elucidate the underlying molecular mechanisms, to evaluate pharmacokinetics and long-term safety, and to assess the translational applicability of this nanoscale delivery system in clinical settings.

## Figures and Tables

**Table 1:** The ferric reducing antioxidant power of CS-based NPs (CS-Pio NPs, CS-Cur NPs, CS-Pio-Cur NPs), APIs (Pio, Cur, Pio-Cur), and ascorbic acid, as a reference standard.

Sample	Concentration (µg/mL)	Reducing Power (%)	IC_50_ (µg/mL)
Ascorbic acid	0.18	39.0 ± 0.3	0.28 ± 0.01
	0.30	50.0 ± 0.1	
	0.35	60.0 ± 0.7	
Cur	25.00	30.0 ± 0.6	59.54 ± 0.20 ****
	48.33	50.0 ± 0.6	
	73.33	51.0 ± 0.4	
CS-Cur NPs	25.00	40.0 ± 0.6	29.59 ± 0.09 ### ****
	48.33	77.0 ± 0.6	
	73.33	85.0 ± 0.5	
Pio	3.33	0.8 ± 0.1	276.62 ± 1.15 ****
	6.66	1.5 ± 0.2	
	10.00	2.1 ± 0.1	
CS-Pio NPs	3.33	2.5 ± 0.2	200.57 ± 0.91 ### ****
	6.66	3.9 ± 0.1	
	10.00	4.1 ± 0.3	
Pio-Cur	3.33/25.00	35.0 ± 0.3	46.34 ± 0.16 ****
	6.66/48.33	61.0 ± 0.2	
	10.00/73.33	73.0 ± 0.3	
CS-Pio-Cur NPs	3.33/25.00	47.0 ± 0.2	25.39 ± 0.23 ### ***
	6.66/48.33	89.0 ± 0.4	
	10.00/73.33	99.0 ± 0.3	

The results are expressed as mean ± SD (*n* = 3). Statistical analysis: *** *p* < 0.001, **** *p* < 0.0001 compared with ascorbic acid; ### *p* < 0.001—compared with APIs.

**Table 2:** Hematological parameters of diabetic rats (Group 1), diabetic rats treated with CS-Pio NPs (Group 3), CS-Cur NPs (Group 4), CS-Pio-Cur NPs (Group 5), Pio (Group 6), Cur (Group 7), Pio-Cur (Group 8), compared with non-diabetic rats (Group 2).

Parameter	Group 1	Group 2	Group 3	Group 4	Group 5	Group 6	Group 7	Group 8
WBC (×10^3^/µL)	10.6 ± 0.2 *	6.3 ± 0.7	8.6 ± 0.9 **	8.2 ± 0.7 **	7.0 ± 0.8 **	10.4 ± 0.6 **	9.5 ± 1.0 **	8.7 ± 0.9 **
RBC (×10^6^ µL)	8.9 ± 1.1 *	5.3 ± 0.4	7.8 ± 1.0 **	7.7 ± 0.9 **	6.1 ± 0.5 **	7.5 ± 0.6 **	7.8 ± 0.9 **	6.7 ± 1.0 **
HGB (g/dL)	8.4 ± 0.7 *	13.9 ± 1.5	13.1 ± 1.2	14.2 ± 1.5	14.1 ± 1.0	12.5 ± 0.6 **	13.8 ± 1.1	13.7 ± 1.0
HCT (%)	46.0 ± 0.6 *	39.1 ± 0.7	41.9 ± 1.0 **	42.6 ± 0.4 **	40.5 ± 0.2 **	44.2 ± 0.2	45.7 ± 0.9	43.5 ± 1.1
MCV (fL)	51.5 ± 1.0 *	72.0 ± 1.4	60.8 ± 0.7 **	59.7 ± 0.3 **	68.8 ± 1.2 **	59.2 ± 1.1 **	58.4 ± 1.1 **	59.7 ± 1.1 **
MCHC (g/dL)	35.2 ± 0.4 *	34.3 ± 0.8	36.4 ± 0.6 **	33.8 ± 0.4 **	34.8 ± 0.7 **	35.3 ± 0.7	34.1 ± 0.6 **	33.2 ± 0.6 **
RDW-SD (fL)	29.0 ± 0.2 *	37.4 ± 1.0	34.1 ± 1.3 **	32.5 ± 1.5 **	34.6 ± 1.0 **	33.2 ± 0.8 **	31.7 ± 1.5 **	33.1 ± 1.3 **
RDW-CV (%)	22.1 ± 1.7 *	16.2 ± 1.0	18.8 ± 0.7 **	18.1 ± 0.9 **	17.1 ± 1.1 **	18.6 ± 1.9 **	19.8 ± 1.6 **	19.1 ± 0.7 **
PLT (10^3^/µL)	700.0 ± 4.0 *	345.0 ± 4.3	575.0 ± 8.3 **	547.0 ± 9.2 **	488.0 ± 6.0 **	599.0 ± 10.0 **	576.0 ± 12.0 **	580.0 ± 11.2 **
P-LCR (%)	28.8 ± 1.1 *	13.7 ± 1.2	15.5 ± 1.6 **	10.4 ± 1.0 **	9.1 ± 1.1 **	13.4 ± 1.0 **	12.3 ± 0.8 **	11.3 ± 0.6 **
LYM (%)	81.4 ± 0.8 *	60.7 ± 1.3	72.1 ± 1.8 **	73.1 ± 1.4 **	68.2 ± 1.3 **	75.5 ± 2.0 **	75.9 ± 2.0 **	69.3 ± 2.5 **
LYM# (10^3^/µL)	2.9 ± 1.9 *	9.3 ± 1.0	5.4 ± 0.7 **	7.2 ± 0.6 **	8.5 ± 0.7 **	8.5 ± 0.3 **	6.0 ± 0.9 **	6.2 ± 1.0 **
MONO (10^3^/µL)	0.90 ± 0.08 *	0.10 ± 0.01	0.42 ± 0.03 **	0.75 ± 0.04 **	0.38 ± 0.01 **	0.49 ± 0.01 **	0.61 ± 0.02 **	0.55 ± 0.05 **

The results are expressed as mean ± SD (*n* = 5). Statistical analysis: * significant difference compared with Group 2, ** significant difference compared with Group 1 (*p* < 0.05).

**Table 3:** Lipid profile parameters of diabetic rats (Group 1), diabetic rats treated with CS-Pio NPs (Group 3), CS-Cur NPs (Group 4), CS-Pio-Cur NPs (Group 5), Pio (Group 6), Cur (Group 7), Pio-Cur (Group 8), compared with non-diabetic rats (Group 2).

Parameter	Group 1	Group 2	Group 3	Group 4	Group 5	Group 6	Group 7	Group 8
TG (mg/dL)	178.0 ± 2.5 *	72.0 ± 1.1	99.0 ± 1.8 **	103.0 ± 2.2 **	89.0 ± 2.1 **	105.0 ± 1.9 **	115.0 ± 4.2 **	109.0 ± 3.3 **
TC (mg/dL)	115.0 ± 8.2 *	68.0 ± 3.6	88.0 ± 4.2 **	85.0 ± 4.9 **	71.0 ± 3.5 **	90.0 ± 3.0 **	92.0 ± 5.6 **	85.0 ± 4.1 **
VLDL (mg/dL)	37.0 ± 0.7 *	21.0 ± 1.1	25.0 ± 1.6 **	27.0 ± 1.5	18.0 ± 2.2 **	20.0 ± 0.9 **	22.0 ± 0.8 **	19.0 ± 1.0 **
LDL (mg/dL)	48.0 ± 1.8 *	34.0 ± 0.9	38.0 ± 0.8 **	40.0 ± 1.0 **	37.0 ± 1.3 **	41.0 ± 1.9	41.0 ± 2.0	39.0 ± 1.9 **
HDL (mg/dL)	23.0 ± 2.1 *	38.0 ± 2.1	26.0 ± 4.1	28.0 ± 2.3 **	37.0 ± 3.4 **	31.0 ± 2.3 **	27.0 ± 2.2 **	30.0 ± 3.1 **

The results are expressed as mean ± SD (*n* = 5). Statistical analysis: * significant difference compared with Group 2, ** significant difference compared with Group 1 (*p* < 0.05).

**Table 4:** Liver function parameters of diabetic rats (Group 1), diabetic rats treated with CS-Pio NPs (Group 3), CS-Cur NPs (Group 4), CS-Pio-Cur NPs (Group 5), Pio (Group 6), Cur (Group 7), Pio-Cur (Group 8), compared with non-diabetic rats (Group 2).

Parameter	Group 1	Group 2	Group 3	Group 4	Group 5	Group 6	Group 7	Group 8
AST (U/L)	469.0 ± 8.9 *	176.0 ± 10.5	249.0 ± 3.3 **	281.0 ± 2.9 **	213.0 ± 3.3 **	298.0 ± 2.3 **	309.0 ± 2.6 **	289.0 ± 2.9 **
ALT(U/L)	144.0 ± 1.9 *	62.0 ± 0.9	119.0 ± 2.3 **	122.0 ± 1.2 **	96.0 ± 1.7 **	133.0 ± 1.4 **	126.0 ± 1.3 **	125.0 ± 1.6 **
LDH (U/L)	470.0 ± 26.6 *	177.0 ± 9.0	283.0 ± 9.5 **	290.0 ± 11.4 **	216.0 ± 9.0 **	305.0 ± 8.3 **	287.0 ± 10.4 **	279.0 ± 11.1 **
TB (mg/dL)	1.8 ± 0.3 *	0.8 ± 0.1	1.6 ± 0.7 **	1.6 ± 0.7 **	1.1 ± 0.5 **	1.7 ± 1.0 **	1.5 ± 2.0 **	1.7 ± 2.2 **
DB (mg/dL)	0.67 ± 0.01 *	0.15 ± 0.02	0.48 ± 0.04 **	0.42 ± 0.02 **	0.31 ± 0.02 **	0.46 ± 0.03 **	0.42 ± 0.03 **	0.41 ± 0.05 **

The results are expressed as mean ± SD (*n* = 5). Statistical analysis: * significant difference compared with Group 2, ** significant difference compared with Group 1 (*p* < 0.05).

**Table 5:** Renal function parameters of diabetic rats (Group 1), diabetic rats treated with CS-Pio NPs (Group 3), CS-Cur NPs (Group 4), CS-Pio-Cur NPs (Group 5), Pio (Group 6), Cur (Group 7), Pio-Cur (Group 8), compared with non-diabetic rats (Group 2).

Parameter	Group 1	Group 2	Group 3	Group 4	Group 5	Group 6	Group 7	Group 8
Creatinine (mg/dL)	1.21 ± 0.06 *	0.46 ± 0.03	0.72 ± 0.04 **	0.94 ± 0.05 **	0.62 ± 0.04 **	0.70 ± 0.03 **	1.02 ± 0.06 **	0.76 ± 0.05 **
BUN (mg/dL)	96.0 ± 1.9 *	30.0 ± 0.9	64.0 ± 1.3 **	70.0 ± 2.0 **	44.0 ± 1.7 **	73.0 ± 1.5 **	81.0 ± 1.6 **	65.0 ± 1.7 **
Uric acid (mg/dL)	4.64 ± 0.66 *	1.71 ± 0.15	2.97 ± 0.54 **	3.61 ± 0.61 **	2.36 ± 0.36 **	3.45 ± 0.61 **	3.86 ± 1.01 **	2.90 ± 0.93 **

The results are expressed as mean ± SD (*n* = 5). Statistical analysis: * significant difference compared with Group 2, ** significant difference compared with Group 1 (*p* < 0.05).

## Data Availability

The original contributions presented in the study are included in the article; further inquiries can be directed to the corresponding author.
